# Communication dynamics in the care of preterm infants: a multi-level qualitative study from a high-burden district in Indonesia

**DOI:** 10.1186/s12939-026-02914-y

**Published:** 2026-06-05

**Authors:** Mira Miraturrofi’ah, Qorinah Estiningtyas Sakilah Adnani, Dwi Agustian, Lisa McKenna, Dany Hilmanto

**Affiliations:** 1 Doctoral Study Program in Medical Science, Faculty of Medicine, Universitas Padjadjaran, Bandung, Indonesia; 2 Department of Midwifery, Universitas Bhakti Kencana, Bandung, Indonesia; 3 Department of Public Health, Faculty of Medicine, Universitas Padjadjaran, Bandung, Indonesia; 4 School of Nursing and Midwifery, La Trobe University, Bundoora, VIC 3086 Australia; 5 Department of Child Health, Faculty of Medicine, Universitas Padjadjaran, Bandung, Indonesia

**Keywords:** Infant, Preterm, Health communication, Family-centred care, Continuity of patient care, Qualitative research

## Abstract

**Background:**

Preterm birth remains a major contributor to infant mortality and long-term morbidity globally, with disproportionate burdens in low- and middle-income countries, including Indonesia. Global maternal and newborn health agendas increasingly promote continuity of care, family-centred approaches, and behaviour-informed communication across levels of service delivery. However, how globally endorsed principles are translated into routine communication during hospital-to-home transitions is shaped by local health systems, provider capacity, and household contexts, and remains insufficiently understood in high-burden settings. This study aimed to map communication dynamics in preterm infant care in a high-burden district in Indonesia using a multi-level qualitative approach.

**Methods:**

This exploratory multi-level qualitative study applied behavioural and communication frameworks to examine communication processes across clinical, household, and systemic levels in Bandung District, a high-burden area in West Java, involving referral hospitals and community health facilities. Purposive sampling included 59 participants, comprising mothers of preterm infants, hospital and primary health care providers, district and provincial health authorities, professional groups, and the national health insurance administrators. Data were gathered via in-depth interviews, focus group discussions, participant observations, and document analysis. Reflexive Thematic Analysis was conducted with NVivo 15, incorporating iterative coding, memoing, cluster analysis, and theme refinement. Rigour was maintained through triangulation, audit trails, and peer debriefing.

**Result:**

Four interconnected themes encapsulated the communication landscape: Communication Practices (channels, techniques, timing, message content, interprofessional handover); Household and Community Support (health literacy, practical skills, beliefs, motivation/self-efficacy, emotional coping, family and peer support); Worker Capacity and Resources (training deficiencies, workload/time constraints, educational materials, supervision, standard operating procedures); and System, Policy, and Access (policy, monitoring, financing, interagency coordination, referral pathways, infrastructure, geographic/economic accessibility). These themes collectively demonstrate how fragmented practices, inconsistent provider capability, and varying family preparedness produce inequitable continuity of care, hindering effective transitions from hospital to home.

**Conclusion:**

Communication in preterm infant care is influenced by interrelated interpersonal, household, workforce, and systemic factors. Strengthening continuity of care requires integrated, multi-tiered strategies that align communication practices across levels of service delivery to reduce inequities in post-discharge care and prevent avoidable neonatal risks in high-burden settings.

**Supplementary Information:**

The online version contains supplementary material available at 10.1186/s12939-026-02914-y.

## Background

Preterm birth (birth prior to 37 weeks) continues to be the primary cause of infant mortality and a significant factor in the global disease burden [1–6]. In 2020, the WHO and UNICEF estimated the global preterm birth rate to be between 4% and 16%, impacting approximately 13–14 million infants [1]. Complications arising from prematurity are the primary cause of mortality in children under five, showing minimal global reduction, and significantly contribute to long-term morbidity and Disability-Adjusted Life Years (DALYs) [7]. Post-discharge readmission rates exhibit significant variability, indicating diverse risk factors and the necessity for tailored interventions [8–15].

The necessity to enhance care for preterm infants in Indonesia is evident. The national prevalence of preterm birth surpasses 10%, resulting in hundreds of thousands of instances each year and significantly impacting infant mortality [16, 17]. Neonatal mortality continues to be elevated in comparison to neighbouring Southeast Asian countries, with West Java bearing a significant burden [16]. Multiple districts within the Bandung region are designated as high-burden areas for infant mortality, while rates of preterm birth continue to be significantly elevated [6, 17–21]. Operational limitations regarding referral capacity and continuity of care at Primary Health Care (PHC) facilities and private midwife practices (PMB) render the post-hospital transition a vital factor in determining long-term outcomes [22]. These geographic and systemic constraints reflect persistent health inequities, whereby families residing in high-burden districts experience systematically reduced access to coordinated post-discharge support and continuity of care compared to populations in better-resourced settings.

The transfer of care for preterm infants from hospital to home represents a vital phase which parents can encounter deficiencies in knowledge, self-efficacy, and emotional preparedness. These disparities affect home-based care practices, developmental and growth monitoring, and the likelihood of readmission. Cross-level communication—connecting hospital perinatal units, primary healthcare services, and families—is vital for ensuring continuity of post-discharge care [23–25], yet is frequently unsynchronised, resulting in fragmented information and inconsistent support. Such communication failures do not affect all families equally; rather, they disproportionately disadvantage caregivers with limited health literacy, social support, and access to follow-up services. As a result, communication functions as a structural mechanism through which inequities in caregiving capacity and infant health outcomes are produced and reinforced. In numerous low and middle-income countries (LMICs), the absence of structured communication frameworks that facilitate integrated services across different levels of care continues to hinder parental preparedness and diminishes the continuity between hospital guidance and community follow-up care. Evidence indicates that discharge education is frequently inconsistent, pre-discharge communications are inadequately organised, and community follow-up procedures are not consistently focused on ongoing education and practical support [23, 24, 26]. These deficiencies hinder parental cognitive, behavioural, and psychological preparedness, elevating the risks of inadequate home care and readmission. Informal tools, such as instant messaging (e.g., WhatsApp), are occasionally used to address communication deficiencies; however, they are unstructured, non-educational, and inadequate for ensuring continuity of care [27–29]. While such informal approaches may temporarily benefit some families, their unstructured and exclusionary nature risks further widening inequities in access to reliable, consistent, and actionable caregiving information. The WHO emphasises that reducing issues associated with preterm necessitates effective communication, especially Behaviour Change Communication (BCC) [7, 30]. Although educational and BCC strategies enhance family knowledge and in-home care [30–33], key gaps remain.

Over the past two decades, global maternal and newborn health agendas have increasingly emphasised continuity of care, family-centred approaches, and behaviour-informed communication across levels of service delivery. These priorities, articulated through international guidelines and professional standards, are expected to be operationalised within national and subnational health systems. However, how such globally informed principles are interpreted and enacted in routine communication practices, particularly during the transition from hospital to home in high-burden settings, remains insufficiently understood [34]. Uneven translation of these globally endorsed principles into routine practice contributes to inequitable realisation of care standards across health system levels, particularly in resource-constrained and high-burden contexts. No communication model has been explicitly developed for preterm infant care that incorporates family-centred care concepts and ensures continuity across hospital, primary care, and community levels [27, 35]. Secondly, numerous studies concentrate exclusively on unidirectional clinical instruction and fail to delineate the multifaceted factors—encompassing personal, household, provider, and systemic domains—that influence communication processes [28, 35]. Third, explicit assessments based on behaviour-change theory are limited in preterm infant care within LMICs [27]. These deficiencies hinder the development of comprehensive interventions designed to prevent complications and reduce readmission rates.

This study sought to map the communication dynamics in preterm infant care within a high-burden district in Indonesia through a multi-level qualitative study, and to examine how these dynamics reflect the localisation of globally informed maternal and newborn care principles across multiple levels of the health system, with the objective of informing the creation of an integrated, behaviour-informed communication model that enhances family preparedness, service continuity, and equity in preterm infant care.

## Methods

### Study design

This research utilised a multi-tiered exploratory qualitative framework to delineate communication patterns in the care of preterm infants. This study was informed by an integrated set of behavioural and health communication frameworks, including the Integrated Behavioral Model (IBM), Health Belief Model (HBM), Social Cognitive Theory (SCT), Social and Behavioral Change Communication (SBCC), and the Continuum of Care framework. These frameworks were operationalised to guide the development of data collection instruments and to inform the analytical focus across individual, interpersonal, community, and system levels.

Constructs from IBM, HBM, and SCT were used to explore caregivers’ knowledge, beliefs, perceived risks and benefits, self-efficacy, and social support related to preterm infant care. SBCC principles guided the examination of communication practices, message clarity, timing, and repetition, while the Continuum of Care framework informed the exploration of communication continuity and fragmentation across facility-based and community-based services. These frameworks functioned as sensitising conceptual scaffolds rather than prescriptive coding schemes, allowing themes to emerge inductively from the data. Data were analysed using Reflexive Thematic Analysis, following Braun and Clarke’s six-step approach [36].

### Study setting

The study was conducted in Bandung District, West Java, an area with a substantial burden of infant mortality. West Java reports higher preterm birth rates than neighbouring provinces, and Bandung District consistently records infant mortality rates above the provincial average, with low birth weight and prematurity accounting for approximately 54.98% of infant deaths.

The study involved two referral hospitals with established perinatology services—Otto Iskandar Dinata District Hospital and Welas Asih Provincial Hospital—which receive a high volume of preterm infant referrals. To capture communication dynamics across the referral pathway, nine primary health centres with the highest referral contributions to these hospitals were also included.

The reporting of this qualitative study was guided by the Consolidated Criteria for Reporting Qualitative Research (COREQ) checklist, (see Additional file 1).

### Participants and sampling

A total of 59 participants were recruited via purposive sampling until theoretical saturation was attained [37, 38]. Potential participants were identified through coordination with leadership at referral hospitals, primary health centres, and relevant health system institutions, including district and provincial health offices responsible for maternal and child health and health promotion programmes, the National Health Insurance Administrator (NHIA), and relevant professional organisations. Leadership involvement was limited to facilitating access to eligible personnel rather than determining participant selection. Participants were approached in person and invited through formal written correspondence. Eligibility was independently verified by the research team based on predefined inclusion criteria requiring direct participation in communication along the preterm care continuum. Recruitment across multiple institutions, professional roles, and levels of care was undertaken to ensure diverse perspectives and minimise potential selection bias.

Participants comprised five categories: mothers of preterm infants; hospital-based providers (heads of NICU/perinatology, paediatricians, nurses); primary health care providers (heads of PHC, midwives, health cadres); representatives from district and provincial health offices; and professional organisations alongside the National Health Insurance Administrator (NHIA). The exclusion criteria encompassed significant congenital defects, communicative impediments, or refusal to engage. No invited participants declined or retracted their participation.

### Data collection

Prior to data collection, the guidelines for the in-depth interviews (IDIs) and focus group discussions (FGDs) were piloted with representatives from all informant groups to ensure clarity and relevance. (see Additional file 2). Interview and focus group guides were developed to reflect key theoretical domains, including caregiver knowledge, beliefs, self-efficacy, social support, and experiences of communication across levels of care, while retaining flexibility to explore emergent issues.

The development of the guides was informed by behavioural change and health communication frameworks, with domains mapped across individual, interpersonal, community, and health system levels. This structure enabled exploration of caregiver readiness and self-efficacy, family and peer support, health worker communication practices, and system-level coordination and continuity of care. Although theory-informed, all questions were open-ended and adaptively probed to allow unanticipated issues and meanings to emerge from participants’ narratives.

Primary data were collected via in-depth interviews, focus group discussions, participant observations, and document analysis. In-depth interviews with mothers, paediatricians, and key informants examined communication experiences during hospital treatment and the transition to home.

Focus group discussions with primary healthcare providers, hospital personnel, and health authorities including representatives from the NHIA - captured perspectives on communication practices, coordination, and referral processes across community, primary care, hospital, and health system governance levels. Observations during discharge planning, breastfeeding sessions, and kangaroo care recorded real-time communication methodologies. Two nurses engaged in focus group discussions and were also incorporated into normal care observations during this process, with no non-participants present.

All in-depth interviews (IDIs) and focus group discussions (FGDs) lasting 30 to 120 min, along with observations lasting 20 to 40 min, were conducted in Indonesian from August to November 2025 by two members of the research team (MM and QESA); during the focus group discussions, an additional research assistant served as a note-taker. These sessions were audio-recorded with written consent and transcribed verbatim in Indonesian by the first author (MM), supplemented with field notes, and verified for accuracy before analysis. Data analysis was conducted in Indonesian to preserve participants’ original meanings. For reporting purposes, selected quotations were translated into English and reviewed by bilingual members of the research team (QESA and DA) to ensure accuracy and fidelity to the original transcripts.

At the conclusion of each interview and focus group discussion, the moderator summarised the salient points and encouraged participants to explain, augment, or amend their remarks as part of real-time member verification to enhance interpretive credibility; all participants affirmed the accuracy without requesting alterations. Full transcripts were not returned to participants, as verification occurred during the sessions through immediate participant confirmation.

No prior relationship existed, and the research objectives were articulated clearly to all participants. Participants were informed that the interviewer was a midwifery lecturer and doctoral researcher conducting the study for academic research purposes. The interviewer’s professional background in maternal and newborn care was acknowledged as a potential influence on data collection and interpretation, and reflexive awareness was maintained throughout the research process.

### Data analysis

Qualitative analysis adhered to Braun and Clarke’s six-phase Reflexive Thematic Analysis [36]. The analysis was conducted by two members of the research team (MM and QESA). An inductive coding process was applied across the dataset to identify patterns and generate themes. Behavioural and communication theories were used as sensitising frameworks to guide analytic focus during the later stages of theme refinement and interpretive synthesis rather than during the initial coding process. NVivo 15 supported data management and iterative reflexive coding. Consistent with the principles of reflexive thematic analysis, numerical intercoder reliability was not applied [36].

#### Familiarisation with data 

All interview, FGD, observation, and document transcripts were reviewed to build data immersion. Transcripts were verified through audio files, cleaned, and repeatedly read to identify linguistic patterns and in-vivo terms. Word-frequency queries supported initial data familiarisation (see Additional file 3), and a word cloud was used to summarise frequently occurring terms (see Additional file 4).

#### Generating initial codes

Inductive coding was applied across the full dataset. Relevant excerpts were systematically assigned to NVivo nodes with defined labels and in-vivo references. This process generated 52 initial codes spanning communication channels, techniques, message content, knowledge, financing, and referral processes (see Additional file 5). Through iterative review and semantic reflection, overlapping codes were consolidated into broader categories, resulting in 26 refined codes (see Additional file 6). Coding relationships were further examined using cluster analysis by coding similarity (see Additional file 7), and coding decisions were revisited reflexively to ensure alignment with participants’ meanings.

#### Searching for themes

Codes were examined to identify broader semantic and conceptual patterns, following the principles of internal homogeneity and external heterogeneity. Communication-related codes formed the preliminary theme Communication Practice, while other codes formed Family Knowledge and Beliefs, Social and Emotional Support, Health Worker Capacity and Resources, and System, Policy, and Access (see Additional file 8).

#### Reviewing themes

Potential themes were reviewed against full data extracts, subtheme coherence, and co-occurrence patterns (using the Matrix Coding Query). Peer debriefing informed refinement. The overlap between Family Knowledge and Beliefs and Social and Emotional Support warranted integration into a single theme: Household and Community Support, reflecting both empirical and conceptual cohesion. The structure was refined to four stable themes, (see Additional file 9).

#### Defining and naming themes

Themes were finalised by articulating their conceptual essence, defining subthemes, and developing operational descriptions. The final structure comprised 23 subthemes across four main themes, (see Additional file 10).

#### Producing the report

Themes and subthemes were synthesised using pseudonymised quotations, with interpretations informed by the IBM, HBM, SCT, SBCC, and MCoC frameworks to enhance explanatory depth, while remaining firmly grounded in participants’ accounts [30, 39–42]. Data triangulation was performed by comparing patterns across interviews, focus group discussions, observations, and document analysis to identify convergent and complementary insights across the dataset.

### Rigor and ethics

The research team comprised multidisciplinary professionals in midwifery, public health, medicine, epidemiology, and paediatrics, all of whom were members of their respective national health professional associations, including the Indonesian Midwives Association (Ikatan Bidan Indonesia, IBI), the Indonesian Medical Association (Ikatan Dokter Indonesia, IDI), and the Indonesian Pediatric Society (Ikatan Dokter Anak Indonesia, IDAI). The first author (MM), a female midwife and midwifery lecturer who was a doctoral candidate at the time of the study, and formally trained in qualitative interviewing, reflexive thematic analysis, and the use of NVivo 15, conducted all in-depth interviews, focus group discussions, and observations. The second author (QESA), a female PhD-trained midwifery scholar with extensive experience in qualitative health research, contributed to methodological guidance and analytic rigor. The third author (DA), a male public health physician–epidemiologist (MD), and the fourth author (DH), a male professor of paediatrics (MD, PhD), provided system-level, epidemiological, and clinical expertise. Collectively, the team’s complementary disciplinary backgrounds, professional experience, and gender diversity strengthened reflexivity, methodological rigor, and the robustness of the study’s interpretations.

**Fig. 1:**
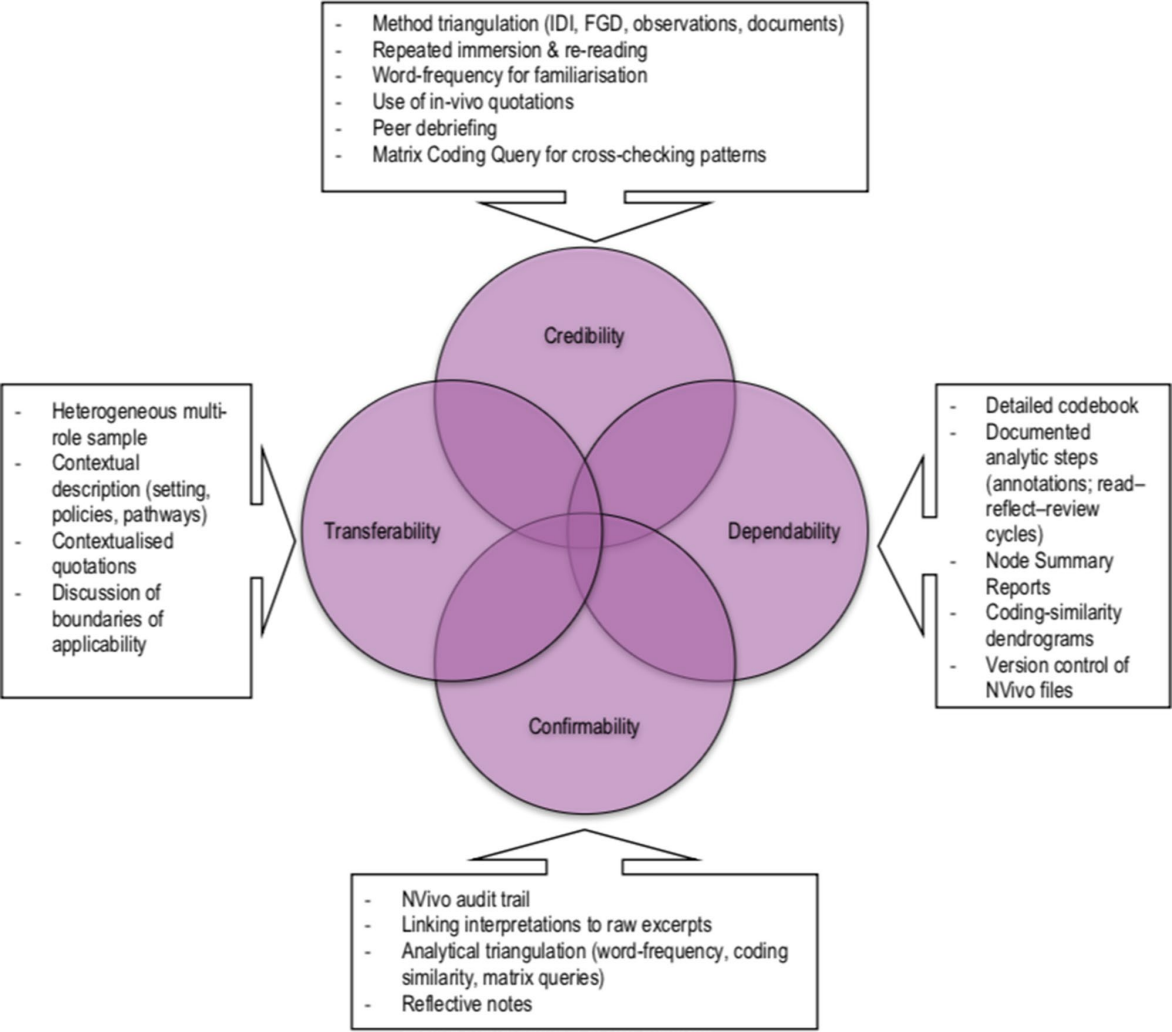
Rigor strategies. This figure illustrates strategies used to ensure rigor in the study

To ensure methodological robustness and analytical quality, four core rigor criteria were applied. An overview of these rigor strategies is presented in Fig. 1.

## Results

### Participant characteristics

Participants represented a range of roles across the maternal–child health system, including mothers of preterm infants, community health cadres, frontline health workers, facility managers, and policy and financing stakeholders. These participants were drawn from family, community, facility, and health system levels of care. Mothers of preterm infants predominantly had low-to-middle educational attainment and varied parity. Community health cadres had substantial years of service in community-based maternal and child health activities. Health system informants included frontline providers, facility managers, representatives from professional organisations, and insurance administrators. Key participant characteristics are summarised in Tables 1, 2 and 3.

**Table 1:** Characteristics of mothers of preterm infants

Informant code	Age (years)	Education	Occupation	Number of children	Gestational age (weeks)
MOTHER_01	40	Primary school	Housewife	3	30
MOTHER_02	30	Junior high school	Housewife	2	32
MOTHER_03	31	Senior high school	Housewife	2	29
MOTHER_04	39	Junior high school	Housewife	3	33
MOTHER_05	37	Senior high school	Housewife	4	34
MOTHER_06	32	Junior high school	Housewife	2	29
MOTHER_07	42	Junior high school	Housewife	2	34
MOTHER_08	29	Junior high school	Housewife	3	36

This table provides contextual information on mothers of preterm infants included in the study

**Table 2:** Characteristics of community health cadre

Informant code	Age (years)	Education	Role	Years of service
COMM-CADRE-01	43	Senior high school	Health cadre	20
COMM_CADRE_02	42	Senior high school	Health cadre	17
COMM_CADRE_03	42	Senior high school	Health cadre	5
COMM_CADRE_04	58	Senior high school	Health cadre	14
COMM_CADRE_05	47	Senior high school	Health cadre	6
COMM_CADRE_06	57	Senior high school	Health cadre	25
COMM_CADRE_07	43	Senior high school	Health cadre	10
COMM_CADRE_08	48	Senior high school	Health cadre	4
COMM_CADRE_09	36	Primary school	Health cadre	3
COMM-CADRE-010	34	Senior high school	Health cadre	3

This table provides contextual information on community health cadres involved in the study

**Table 3:** Characteristics of health system and stakeholder informants. (Hospital, primary care, and government/professional bodies)

Informant code	Age (years)	Role / Category	Profession	Education	Years of experience
HOSP_NUR_01	33	Hospital health worker	Nurse	Professional degree	10
HOSP_NUR_02	44	Hospital health worker	Nurse	Professional degree	25
HOSP_NUR_03	49	Hospital health worker	Nurse	Bachelor’s degree	12
HOSP_NUR_04	26	Hospital health worker	Nurse	Bachelor’s degree	4
HOSP_NUR_05	47	Hospital health worker	Nurse	Professional degree	23
HOSP_PED_01	54	Hospital health worker	Paediatrician	Specialist degree	25
HOSP_NEO_01	50	Hospital health worker	Consultant neonatologist	Sub specialist degree	11
HOSP_NUR_06	29	Hospital health worker	Nurse	Bachelor’s degree	3
COMM_MID_01	44	Community health worker	Midwife	Bachelor’s degree	23
COMM_MID_02	54	Community health worker	Midwife	Professional degree	32
COMM_MID_03	42	Community health worker	Midwife	Professional degree	12
COMM_MID_04	41	Community health worker	Midwife	Bachelor’s degree	19
COMM_MID_05	44	Community health worker	Midwife	Bachelor’s degree	12
COMM_MID_06	55	Community health worker	Midwife	Bachelor’s degree	6
COMM_MID_07	36	Community health worker	Midwife	Professional degree	13
COMM_MID_08	37	Community health worker	Midwife	Bachelor’s degree	15
COMM_MID_09	33	Community health worker	Midwife	Bachelor’s degree	5
PHC_GP_01	41	Unit head (PHC)	General practitioner	Professional degree	5
PHC_GP_02	58	Unit head (PHC)	General practitioner	Professional degree	5
PHC_GP_03	36	Unit head (PHC)	General practitioner	Professional degree	6
PHC_GP_04	52	Unit head (PHC)	General practitioner	Professional degree	6
PHC_GP_05	46	Unit head (PHC)	General practitioner	Professional degree	12
PHC_GP_06	50	Unit head (PHC)	General practitioner	Professional degree	> 3
PHC_GP_07	49	Unit head (PHC)	General practitioner	Professional degree	> 3
PHC_GP_08	44	Unit head (PHC)	General practitioner	Professional degree	6
PHC_GP_09	45	Unit head (PHC)	General practitioner	Professional degree	> 3
HOSP_MGR_NUR_01	45	Unit head (Hospital)	Nurse	Professional degree	7
HOSP_MGR_NUR_02	44	Unit head (Hospital)	Nurse	Professional degree	3
HOSP_MGR_NUR_03	47	Unit head (Hospital)	Nurse	Professional degree	25
SYS_NUR_01	49	Health professional associations	Nurse	Doctoral degree	-
SYS_NEO_01	40	Health professional associations	Consultant neonatologist	Sub specialist degree	-
SYS_MID_01	63	Health professional associations	Midwife	Master’s degree	-
SYS_PHE_01	42	Health professional associations	Public health expert	Doctoral degree	-
SYS_PHE_02	48	Health professional associations	Public health expert	Master’s degree	-
SYS_INS_GP_01	42	NHIA representative	General practitioner	Master’s degree	-
SYS_INS_NUR_01	33	NHIA representative	Nurse	Bachelor’s degree	-
SYS_PROV_GP_01	44	Provincial health office	General practitioner	Master’s degree	-
SYS_PROV_PHE_01	41	Provincial health office	Public health expert	Master’s degree	-
SYS_DIST_GP_01	53	District health office	General practitioner	Master’s degree	-
SYS_DIST_PHE_01	42	District health office	Public health expert	Bachelor’s degree	-
SYS_DIST_MID_01	46	District health office	Midwife	Bachelor’s degree	-

“Bachelor’s degree” refers to completion of an undergraduate academic degree (e.g., Bachelor of Medicine, Bachelor of Midwifery, Bachelor of Nursing)

“Professional degree” refers to the completion of a post-bachelor’s professional education and clinical licensure program, which is required for independent practice in Indonesia (e.g., medical doctor, registered midwife, or registered nurse)

### Core findings

#### Theme 1. Communication practice

This theme captures the operational logic through which communication was enacted across clinical and community settings, illustrating how channel selection, timing, message content, and interprofessional coordination shaped caregivers’ understanding and readiness for home-based preterm infant care.

##### Channels

Direct communication served as the primary and most trusted mode of communication. Participants consistently described face-to-face interaction as the foundation of effective communication across hospital and community care settings, particularly when explaining complex aspects of preterm infant care.

Most communication is done directly. (HOSP_MGR_NUR_01)

You have to meet in person… that’s when trust appears. (HOSP_PED_01)

Continuity was strengthened through home visits—

Information is shared through home visits… (COMM_MID_03).

One mother described how responsive communication from health workers helped her feel supported during home care:

What matters is that someone responds when we ask something. When they answer our questions, it makes us feel calmer. (MOTHER_03)

These accounts suggest that trust and clarity in communication were closely associated to direct interpersonal interaction, especially during the transition from hospital treatment to home-based care.

##### Techniques

Techniques emphasised the use of simple language and attentive listening. Participants highlighted that effective communication required adapting explanations to caregivers’ educational backgrounds and emotional readiness.

The language is too medical… they don’t understand. (SYS_PROV_GP_01 ).

Use local language. (PHC_GP_04)

Active listening was considered foundational: We listen first… don’t rush to give answers. (COMM_MID_02).

Infant condition also guided communication: The baby’s condition… must be clear first. (HOSP_MGR_NUR_01).

Together, these accounts suggest that communication practices involved adapting explanations to caregivers’ comprehension levels and emotional readiness.

##### Timing and dosage

Education was delivered gradually and repeatedly. Participants described communication as an ongoing process rather than a single educational event, particularly for caregivers adjusting to complex neonatal care practices.

If not repeated, the mother won’t understand. (PHC_GP_07)

Timing aligned with clinical readiness: Before discharge… that’s when we plan the return home. (HOSP_MGR_NUR_03).

These narratives suggest that repeated communication supported caregiver understanding and readiness during the transition from hospital care to home management.

##### Message content

Messages covered danger signs, lactation, Kangaroo Mother Care (KMC), thermoregulation, and basic care. Participants emphasised that communication prioritised practical information required for safe home-based care of preterm infants.

Bluish skin, vomiting… danger signs. (HOSP_NUR_04/MOTHER_08)

Families must recognise critical conditions. (PHC_GP_08)

Lactation and KMC were emphasised: “Breast milk is better than formula”. (MOTHER_03); “Kangaroo care must continue”. (COMM_MID_02).

Mothers also described how practical guidance about home care was particularly meaningful after discharge:

They told me not to let the baby get cold… the room should be warm. I always remember that advice when caring for the baby at home. (MOTHER_03)

These accounts show that communication often emphasised translating clinical information into practical caregiving actions for families.

##### Interprofessional handover

Coordination was seen to ensure message consistency. Participants emphasised that communication across professional roles was necessary to maintain consistent messages throughout the care pathway. Nursing staff focused on conveying information related to nursing care, while medical issues remained under the authority of the attending physician, thereby helping prevent contradictory or overlapping messages to families.

What we conveyed was limited to the nursing care we provided. Matters related to disease or other medical issues were rarely communicated by us because those were under the authority of the attending physician. (HOSP_MGR_NUR_02)

Overall, the findings in this theme suggest that communication practices involved multiple interacting elements, including message content, timing, delivery approaches, and coordination among health professionals during the transition from hospital to home care. This pattern is illustrated in the visualisation of inter-subtheme relationships within the Communication Practice theme (see Additional file 11).

#### Theme 2. Household and community support

This theme highlights how family capacity, sociocultural beliefs, emotional readiness, and community support shape home-based preterm care.

##### Knowledge and practical skills

Knowledge varied with education, experience, and parity. Participants described substantial variation in caregivers’ baseline knowledge and caregiving skills, shaping how communication messages were understood and applied.

Sometimes communication doesn’t connect… most only completed junior high school. (SYS_PROV_GP_01 ; PHC_GP_07)

Parity appeared to help, but experience did not always guarantee competence:

For first-time mothers… I explain slowly. (COMM_CADRE_10)

Even experienced mothers forget correct breastfeeding positions. (HOSP_NUR_03)

These accounts suggest that communication strategies needed to accommodate varying levels of caregiver knowledge and prior experience.

##### Beliefs and myths

Cultural norms and authority figures influenced message acceptance. Participants noted that sociocultural beliefs and trusted community figures often shaped how medical advice was interpreted.

In remote areas… they obey information from religious leaders more. (SYS_PROV_GP_01 )

Clashes happen… families believe in blessed water or think jaundice is normal. (HOSP_MGR_NUR_02)

##### Motivation and self-efficacy

Family motivation fluctuated; some expressed fear or resignation.

One mother expressed uncertainty about her ability to care for her premature baby at home:

I was confused… when they said the baby could go home, I kept wondering, can I really take care of the baby? (MOTHER_04)

Participants described caregiver motivation as dynamic, often influenced by emotional stress and perceived caregiving difficulty.

Some families give up first. (PHC_GP_05)

Afraid they cannot manage. (PHC_GP_09)

Persistence was seen to improve outcomes:

Because the mother was diligent… Alhamdulillah the baby became healthy. (COMM_CADRE_04)

These narratives indicate that sustained motivation and confidence were critical for maintaining caregiving practices at home.

##### Emotional state and coping

Anxiety reportedly hindered information uptake. Health professionals frequently described encountering emotional distress among caregivers during hospitalisation and discharge preparation.

Anxiety, fear, worry… we encounter these most often. (HOSP_MGR_NUR_02)

Support eased distress:

After explanation… slowly they understood… became calmer. (COMM_CADRE_03)

Some reported experiencing denial:

Still in denial, unable to accept the baby’s condition. (SYS_NUR_01)

These findings suggest that emotional readiness influenced how caregivers processed and applied health information.

Some mothers also described actively seeking clarification from health workers to reduce uncertainty:

I kept asking questions—how to bathe the baby, how about the immunisations—because I wanted to be sure I was doing it correctly. (MOTHER_04).

##### Family support role

Partner and family involvement was seen to strengthen care decisions.

We try to ensure the husband is present for psychological support. (COMM_MID_03)

However, family hierarchy also influenced decision-making:

Parents don’t have the final say… the grandmother intervenes. (HOSP_NUR_05)

These accounts suggest that family authority structures influenced how caregiving practices were negotiated and implemented.

##### Community/peer support

Community health cadres, village midwives, and Family Assistance Teams (Tim Pendamping Keluarga, TPK) were involved in maintaining follow-up communication with families.

We involve community health cadres to keep communication with families. (PHC_GP_08)

Taken together, these findings indicate that household and community factors influenced how clinical information was interpreted, prioritised, and enacted in home-based preterm care. Family hierarchies, emotional readiness, and community support structures function as critical filters that determine whether communication translates into sustained caregiving practices. These interconnections are depicted in the visualisation of inter-subtheme relationships within the Household and Community Support theme (see Additional file 12).

#### Theme 3. Worker capacity and resources

This theme reflects competencies, workload, educational tools, supervision, and protocols shaping communication quality.

##### Training and skills

Training formed the competency foundation. Participants described professional training as the primary mechanism through which health workers developed communication competencies for supporting families of preterm infants.

We provide various trainings… paediatric and maternal nursing… (SYS_NUR_01)

Effective communication training existed but was not sustained (HOSP_MGR_NUR_01)

Uneven access was perceived to create skill disparities. These accounts suggest that training contributed to baseline communication competencies, although access and continuity varied across settings.

##### Workload and time constraints

Responsiveness was seen to compensate for time limitations. Health professionals consistently reported that heavy workloads limited the time available for comprehensive caregiver education, constraining the depth and consistency of communication with families.

We have a dedicated WhatsApp group… they can ask directly. (COMM_MID_01 )

If they need anything, I’m in the next room. (HOSP_NUR_06)

High workload was reported to reduce time for thorough education. These strategies demonstrate how health workers adapted communication practices to maintain responsiveness despite structural time constraints.

##### Educational materials

Audiovisual media were reportedly preferred. Health professionals reported that educational tools played an important role in reinforcing verbal communication with caregivers.

More effective through audiovisual. (HOSP_MGR_NUR_02)

However, low use of the Maternal and Child Health (MCH) handbook and the absence of dedicated communication teams were reported to hinder consistency.

##### Supervision and evaluation

Supervision was described as occurring via routine evaluations and direct observation:

We evaluate every activity… (PHC_GP_04)

I often observe how staff communicate. (HOSP_MGR_NUR_01)

These practices indicate that ongoing supervision supported reflective improvement in communication practices among health workers.

##### SOPs and protocols

Lack of standard communication SOPs emerged as a major gap:

No standardised SOP [standard operating procedures ]… it needs to be developed.(HOSP_PED_01)

Collectively, these findings suggest that health-worker communication capacity was shaped by the interplay between individual skills, workload pressures, and the availability of institutional support systems, highlighting that communication practices were influenced not only by individual training, but also by organisational resources and institutional support systems. These inter-subtheme relationships are illustrated in the visualisation of the Worker Capacity and Resources theme (Supplementary File 13).

#### Theme 4. System, policy, and access

Communication effectiveness was shaped by policy frameworks, multisectoral coordination, financing, referral systems, resource availability, and geographic access.

##### Policy and regulation

Prematurity-specific communication guidelines were reported to be absent.

In health… nothing specific for preterm infants. (SYS_PHE_01)

For prematurity specifically… none yet. (SYS_DIST_GP_01)

##### Programme evaluation (monitoring and evaluation, monev)

Monitoring reportedly remained inconsistent due to the limited availability of indicators and human resources. Participants described evaluation mechanisms as fragmented, with limited tools for systematically assessing communication programmes.

Don’t design a communication programme without measures of success. (SYS_PHE_01)

Cross-facility monitoring was seen to be uneven:

No mechanism besides the control card… (SYS_PROV_GP_01 )

We can’t visit all facilities for monitoring. (SYS_DIST_GP_01)

These constraints suggest that limited monitoring systems affected the ability to assess communication activities across facilities.

##### Financing and NHIA administration

Financial protection was seen to influence continuity of care.

With active NHIA [National Health Insurance Administrator ], it helps a lot… no need to worry. (MOTHER_03)

Without NHIA… referral for preterm infants is very difficult. (COMM_MID_03)

These narratives highlight the role of financial protection mechanisms in sustaining continuity of care after hospital discharge.

##### Interagency coordination

Coordination was seen to ensure consistent messaging. Participants highlighted that collaboration across institutions was necessary to maintain coherent communication throughout the care pathway.

We must stay connected… information from the hospital must reach field workers. (SYS_MID_01)

Capacity-building activities strengthened communication skills:

We gathered the PKRS team… to enhance communication skills. (SYS_PROV_PHE_01)

Hence, collaborative mechanisms helped align communication practices across institutional levels.

##### Referral system

Referral pathways lacked two-way feedback. Participants described referral communication as largely unidirectional, limiting opportunities for follow-up coordination.

Referral from districts uses Sisrute (Integrated Referral Information System), but there’s no referral back. (SYS_NEO_01)

Documentation was considered to be inconsistent: Sometimes nothing is written in the MCH handbook… (PHC_GP_08).

Hospital capacity constraints slowed clinical flow: Preterm quota is very limited. (COMM_MID_06).

These limitations indicate that referral communication was often incomplete, which affected follow-up coordination after discharge.

##### Resources

NHIA service channels were seen to improve accessibility:

Many channels… even via Zoom. (SYS_INS_GP_01)

Yet, lack of dedicated staff reportedly hindered educational media development:

No dedicated staff… so it doesn’t run. (SYS_PROV_PHE_01)

These findings indicate that communication initiatives depended heavily on the availability of organisational resources.

##### Geographic and economic access

Distance and terrain also reportedly limited follow-up:

The location is far… so no one comes. (HOSP_MGR_NUR_03)

Cold regions also risked hypothermia:

In cold areas… afraid the baby will be hypothermic. (HOSP_NUR_03)

Digital access was also seen to be constrained:

In remote areas, even buying Internet data is difficult. (SYS_PROV_PHE_01).

These barriers demonstrate how structural inequalities in access can limit the effectiveness of communication interventions.

Taken together, this theme illustrates that system-level arrangements—particularly referral pathways, financing mechanisms, and monitoring structures—shaped the conditions within which communication and care coordination occurred after hospital discharge, either enabling continuity of care or amplifying fragmentation after discharge. These links are shown in the System, Policy, and Access visualisation (see Additional file 14).

Overall, findings indicate that communication in preterm infant care functioned as a dynamic, multi-level system, with practices, social contexts, workforce capacity, and policies deeply interdependent. Communication was closely associated with family knowledge, caregiving confidence, and continuity of care after discharge. Additional file 15, illustrates how disruptions at any level can cascade, undermining post-discharge care continuity.

## Discussion

This study delineated communication dynamics in preterm infant care across micro, meso, and macro levels in a high-burden region, identifying four themes and multiple subthemes that illustrate the interplay of communication practices, familial and community support, health worker capacity, and systemic factors in shaping information continuity. Effective information transfer arises from these multi-level interactions, rather than from any singular element.

### Integrating empirical findings, global evidence, and mechanisms of change

#### Communication practice

This study identified Communication Practice as the fundamental mechanism for continuity of care for preterm infants in both clinical and community environments. Evidence suggests that the success of communication is not determined by individual elements, but rather by the coordinated interactions among channels, procedures, message content, timing, and interprofessional handovers. The reciprocal relationship between Channels and Techniques demonstrates that the selection of communication modality directly influences the suitability and efficacy of delivery strategies. The strong connection between interprofessional handover and Message Content highlights how provider coordination affects the coherence and completeness of information received by families [14, 43].

These empirical patterns closely correspond with global evidence regarding family-centred care and discharge communication in LMICs, which consistently underscores that effective communication depends on relational continuity, face-to-face interaction, and systematic handover processes rather than isolated informational exchanges [14, 43]. This study’s emphasis on in-person counselling, home visits, and interpersonal trust aligns with global research indicating that direct communication is the most effective method for imparting caregiving skills and enhancing parental confidence post-discharge, especially in resource-limited environments where formal systems may be disjointed [25].

Theoretical elucidations further clarify these conclusions. According to Social Cognitive Theory (SCT), communication methods, including demonstrations, repeated explanations, and teach-back, serve as mechanisms of modelling and reinforcement that enhance carers’ perceived competence and self-efficacy [40–42]. The efficacy of language modification, sympathetic listening, and organised explanations at the communicative level exemplifies ideas from the Elaboration Likelihood Model and framing theory, indicating that customised, emotionally impactful messages improve understanding and memory retention [44, 45]. Collectively, these strategies elucidate how communication practices facilitate the conversion of clinical knowledge into implementable caregiving behaviours in the home environment.

#### Household and community support as an active communication system

The theme of Household and Community Support illustrates that effective communication transcended the health facility, being significantly influenced by familial dynamics, sociocultural attitudes, emotional conditions, and community support systems. Evidence from this study demonstrates interrelations among beliefs and myths, emotional coping mechanisms, knowledge and skills, motivation, and familial responsibilities. Beliefs and myths were discovered to affect emotional responses, either intensifying fear or offering reassurance, whereas emotional stability subsequently influenced families’ ability to assimilate and use caregiving information. Community and peer support appeared as a vital buffer, alleviating stress and bolstering confidence, whereas familial support—especially from partners and extended relatives—enhanced motivation and sustained caregiving practices. These findings align closely with the international literature, which demonstrates that parental health knowledge, emotional preparedness, cultural norms, and social support significantly influence post-discharge outcomes for preterm infants [15, 46]. International research regularly demonstrates that even meticulously crafted discharge education can be ineffective if communication is not maintained within the household and community environment [15, 46]. This study builds upon existing findings by demonstrating that home and community actors operate not only as contextual factors but also as essential elements of the communication system that influence the interpretation, prioritization, and implementation of messages post-discharge.

These household and community dynamics theoretically correspond with both the Health Belief Model (HBM) and Social Cognitive Theory (SCT). The HBM elucidates how carers’ perceptions of vulnerability and severity, influenced by beliefs and emotional states, affect their reaction to signals regarding danger signs and home care [40]. SCT further clarifies how social reinforcement, observational learning, and emotional support from familial and community members augment self-efficacy and perpetuate caregiving practices over time [40–42]. These mechanisms emphasise that effective communication in preterm infant care must be integrated within the social contexts in which caregiving takes place.

From an equity perspective, family-centred communication functions as a corrective mechanism that mitigates disparities in caregiving capacity by tailoring support to families’ social, emotional, and contextual resources.

#### Worker capacity and resources as enabling conditions for communication quality

The theme of Worker Capacity and Resources emphasises the internal health service factors that facilitate or hinder the quality and consistency of communication. Empirical evidence demonstrates significant interdependencies among workload, training and skills, supervision, educational resources, and standard operating procedures. Excessive workload and time limitations restricted opportunities for training and comprehensive communication, but monitoring and performance assessment were crucial in promoting compliance with communication norms. The accessibility of educational resources correlated with training and expertise, as more proficient providers were more adept at utilising audiovisual tools and demonstrations successfully. The findings align with global evidence from LMICs, indicating that the quality of communication is influenced not just by the competency of individual providers but also by institutional support mechanisms, such as moderate workloads, supervision, and access to suitable resources [27]. In this study, health workers often addressed structural deficiencies through personal dedication and informal digital communication, reflecting global reports of adaptive, yet unsustainable, methods that pose risks of inconsistency and exhaustion [47]. Theoretical integration through the Integrated Behavioural Model (IBM) elucidates these tendencies. In the IBM paradigm, the efficacy of providers’ communication is contingent upon human competencies and supportive settings, which encompass time, resources, and organisational backing [30, 39]. The lack of standardised SOPs identified in this study emphasises that training alone cannot maintain communication quality without structural reinforcement—a conclusion strongly corroborated by global implementation science literature and visually represented in the cross-theme interactions illustrated in Additional file 15.

#### System, policy, and access as structural determinants of communication continuity

At the macro level, System, Policy, and Access establish the structural constraints for communication. Results indicate strong connections between policy, resource availability, program monitoring, referral mechanisms, interagency collaboration, and geographic and economic accessibility. Inadequate prematurity-specific rules limited resources and standardisation, while disjointed monitoring and the lack of reciprocal referral feedback hindered information exchange between hospitals and primary care, although robust interpersonal initiatives [42]. These structural constraints result in inequitable continuity of communication, whereby families in high-burden and resource-constrained settings systematically receive less coordinated, timely, and actionable support compared to those in better-resourced contexts. These difficulties exemplify the health systems of LMICs, where financing, referral frameworks, and information systems collectively influence the continuity of care. NHIA enhanced accessibility and alleviated financial burdens; nonetheless, ongoing deficiencies in documentation and feedback continued to undermine follow-up efforts.

Theoretically, these system-level components exemplify IBM’s “enabling environment”, illustrating how policy, money, and infrastructure affect enduring behavioural change [30, 39]. Geographic and economic obstacles further restrict communication interventions. In accordance with BCC literature, digital platforms like as WhatsApp enhanced reach; but, their efficacy depended on integration with formal systems, pedagogical design, and feedback mechanisms [46, 48].

### Communication as a site of localisation of global maternal and newborn health agendas

Taken together, the findings position communication, not merely as a technical or interpersonal activity, but as a critical site where globally promoted maternal and newborn health principles—such as continuity of care, family-centred approaches, and behaviour-informed communication—are interpreted, adapted, and enacted in everyday practice. Rather than being implemented uniformly, these principles are mediated through household dynamics, health worker capacity, and health system arrangements, resulting in variable enactment across levels of care. This perspective shifts the analytical focus from isolated communication gaps to the processes through which global norms are locally negotiated, revealing how fragmentation in communication reflects broader structural and relational constraints within high-burden settings.

### Dynamic interconnections across the four themes

The results indicate that communication in preterm infant care functions as a dynamic, multi-tiered system where the four themes are intricately interconnected, rather than operating independently. The cross-theme interconnection map demonstrates that communication practices—encompassing channels, techniques, timing, and interprofessional handover—significantly influence family knowledge, motivation, and emotional preparedness. Concurrently, household and community factors, including beliefs, emotional coping, family roles, and peer support, modulate and reinforce the interpretation and implementation of messages in home-based care.

**Fig. 2:**
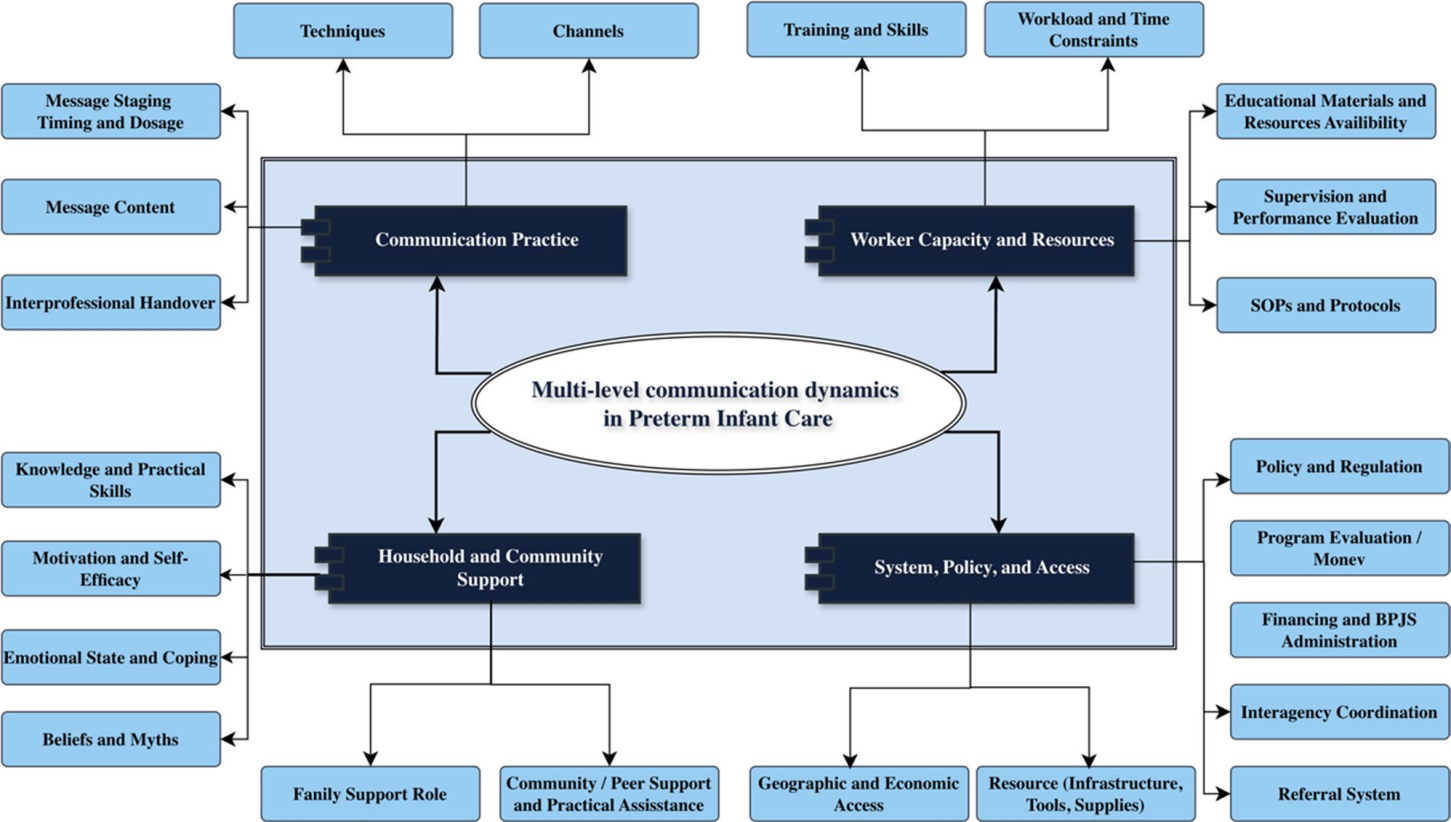
Multi-level communication dynamics in preterm infant care. This figure illustrates the interrelated communication dynamics across individual, household, community, health worker, and system levels that shape continuity of care for preterm infants

The capacity and resources of the workforce serve as a crucial intermediary, connecting frontline communication with systemic issues. Training, supervision, workload, and educational resources affect both the delivery of messages and their uniformity among providers and locations. At the macro level, systemic, policy, and access factors—including referral procedures, financing, monitoring frameworks, and geographic accessibility—establish the structural parameters that facilitate or hinder continuity across the treatment route [39]. These relationships highlight communication as a relational and systemic process that transforms health system inputs into enduring caregiving habits. Effective communication relies on the alignment of family and community participation, supported healthcare professionals, and conducive policy environments, establishing a cohesive framework to enhance family-centred communication and solve continuity gaps post-discharge for preterm infants in low- and middle-income countries [27, 45]. Figure 2 (Multi-Level Communication Dynamics in Preterm Infant Care) illustrates the interrelationships among the identified themes and subthemes, providing a comprehensive view of the dynamic functioning of communication across multiple system levels.

### Policy and practice implications

Addressing communication gaps across practice, system, and policy levels is therefore essential not only for improving care quality, but also for reducing inequities in post-discharge outcomes among preterm infants in high-burden settings. This study presents the subsequent operational recommendations: (1) Standardised discharge communication standard operating procedures for hospitals and primary care, encompassing essential messages, structured referral documentation, and reciprocal feedback; (2) Integrated family communication models incorporating face-to-face counselling, demonstrations, contextual audiovisual tools, and pedagogically designed WhatsApp modules, assessed through implementation studies (knowledge, self-efficacy, readmission) [46, 48]; (3) Competency-based training for healthcare professionals and personnel, using simulation, mentorship, and empathic teach-back and handover methodologies, supported by institutional backing and funding [47, 49, 50]; (4) Two-way referral and digital feedback mechanisms include electronic referrals, reminders, and monitoring indications [51]; and (5) generic BCC materials customised for family readiness, local language, and cultural norms, evaluated for acceptance and comprehension before implementation [40].

Successful execution necessitates dynamic intersectoral cooperation among district health offices, hospitals, primary healthcare facilities, and professional groups, underpinned by continuous funding.

The recommendations serve as adaptive answers to empirically identified deficiencies, establishing conceptual coherence among results, discussion, and implementation strategies to enhance continuity of care and mitigate unnecessary newborn morbidity and mortality.

### Strengths and limitations

The merits of this study are its multi-level design, which captures the dynamic interconnections of communication processes across contexts, rigorous triangulation of methodologies, and the participation of varied informants. Credibility was bolstered by reflexive thematic analysis, NVivo audit logs, and member checking, while theory-informed interpretation augmented policy relevance.

Limitations include the single-district context, potential social desirability bias, and the absence of quantified clinical outcomes.

The findings suggest that communication in preterm infant care operates as a dynamic system, influenced by continuous interactions among various communication practices, family preparedness, healthcare worker competency, and health system integration. Fragmentation at any stage can initiate cascading interruptions, compromising the continuity of care. This discourse illustrates the dynamic alignment of study themes with global data and behaviour-change theory, while establishing a clear connection to the proposed policy and practice implications.

## Conclusion

This study demonstrates that communication in preterm infant care was shaped by the interaction of household contexts, health-worker capacity, and health system arrangements, rather than by isolated communication practices. Evidence from a high-burden district in Indonesia illustrates how globally endorsed maternal and newborn health principles, particularly continuity of care, family-centred approaches, and behaviour-informed communication, are locally interpreted and unevenly operationalised across levels of care, resulting in inequitable continuity of support and fragmented transitions from hospital to home. Strengthening continuity of care therefore requires integrated communication strategies that align family preparedness, provider practices, and system-level coordination to reduce systematic disparities in post-discharge care. Recognising communication as a site where global norms are negotiated in everyday practice is essential for advancing equity and reducing preventable neonatal risks in high-burden settings.

## Electronic Supplementary Material

Below is the link to the electronic supplementary material.

Supplementary Material 1

Supplementary Material 2

Supplementary Material 3

Supplementary Material 4

Supplementary Material 5

Supplementary Material 6

Supplementary Material 7

Supplementary Material 8

Supplementary Material 9

Supplementary Material 10

Supplementary Material 11

Supplementary Material 12

Supplementary Material 13

Supplementary Material 14

Supplementary Material 15

## Data Availability

The qualitative datasets generated and analysed during the current study are not publicly available due to ethical considerations and the need to protect participant confidentiality, but are available from the corresponding author on reasonable request.

## Abbreviations

BCC: Behaviour Change Communication
DALYs: Disability-Adjusted Life Years
HBM: Health Belief Model
IBM: Integrated Behavioral Model
KMC: Kangaroo Mother Care
LMICs: Low- and Middle-Income Countries
MCH: Maternal and Child Health
MCoC: Maternal Continuum of Care
NHIA: National Health Insurance Administrator
NICU: Neonatal Intensive Care Unit
SBCC: Social and Behavioral Change Communication
SCT: Social Cognitive Theory
