# Supplementary material for: Communication dynamics in the care of preterm infants: a multi-level qualitative study from a high-burden district in Indonesia
Source: Int J Equity Health. 2026 Jun 5;25:193. doi: 10.1186/s12939-026-02914-y (PMC13459254; doi:10.1186/s12939-026-02914-y)
Supplement: Supplementary file 1 [file 12939_2026_2914_MOESM10_ESM.pdf]

Supplementary File 8 – Final Themes and Subthemes (Narrative + Tables)

No	Theme	*Definition Essence*	Domain	Subtheme	Number of References	Example Quotation	Implications for the Research Question
1.	*Communication Practice*	The interpersonal and collaborative communication processes between health workers, families, and professional teams in delivering preterm-infant care messages effectively, timely, and with empathy.	Micro	a. *Channels*<br>b. *Techniques*<br>c. *Timing and Dosage*<br>d. *Message Content*<br>e. *Interprofessional Handover*	400 excerpts / 59 participants	*"Most communication is done directly."* (Ka_Ru_Ott_1);<br><br>*"You have to meet in person… that's when trust appears."* (Nks_SPA_Ott_6).<br><br>*"Information is shared through home visits…"* (Nks_Bdn_KIA_3)<br><br>*"Through social media, posters, or short videos."* Ka_Pkm_8<br><br>*The language is too medical… they don't understand."* (EKS_DKJ_3);<br><br>*"Use local language."* (Ka_Pkm_4).<br><br>*"We listen first… don't rush to give answers."* (Nks_Bdn_KIA_2).<br><br>*"The baby's condition… must be clear first."* (Ka_Ru_Ott_1).	Indicates that effective communication success depends not only on message content but also on the techniques employed, the timing of delivery, and the quality of interprofessional coordination.

No	Theme	*Definition Essence*	Domain	Subtheme	Number of References	Example Quotation	Implications for the Research Question
						"Information must be repeated many times." (Ka_Pkm_8);<br><br>*"If not repeated, the mother won't understand."* (Ka_Pkm_7).<br><br>*"Before discharge… that's when we plan the return home."* (Ka_Ru_WA_3).<br><br>*"Bluish skin, vomiting… danger signs."* (Nks_Ners_WA_4/IBP_08);<br><br>*"Families must recognize critical conditions."* (Ka_Pkm_8).<br><br>*"Breast milk is better than formula."* IBP_03<br><br>*"Kangaroo care must continue."* Nks_Bdn_KIA_2<br><br>*"What we convey is limited to nursing care… medical issues are under the attending physician."* (Ka_Ru_Ott_2).	
2.	*Household and Community Support*	The involvement of families and non–health worker social networks in providing emotional, practical, and	Micro–Meso	a. *Knowledge and Practical Skills*	248 excerpts / 59 participants	*"Sometimes communication doesn't connect… most only completed junior high school."* (EKS_DKJ_3; Ka_Pkm_7).	Indicates that social support, family knowledge, and cultural values play a crucial

No	Theme	*Definition Essence*	Domain	Subtheme	Number of References	Example Quotation	Implications for the Research Question
		experiential knowledge–based support that strengthens preterm-infant caregiving practices.		b. *Emotional State and Coping*<br>c. *Motivation and Self-Efficacy*<br>d. *Family Support Role*<br>e. *Beliefs and Myths*<br>f. *Community/Peer Support and Practical Assistance*		*"For first-time mothers… I explain slowly."* (KDR_10);<br><br>*"Even experienced mothers forget correct breastfeeding positions."* (Nks_Ners_Ott_3).<br><br>*"In remote areas… they obey information from religious leaders more."* (EKS_DKJ_3).<br><br>*"Clashes happen… families believe in blessed water or think jaundice is normal."* (Ka_Ru_Ott_2).<br><br>*"Some families give up first." (Ka_Pkm_5)*<br><br>*"Afraid they cannot manage."* (Ka_Pkm_9).<br><br>*"Because the mother was diligent… Alhamdulillah the baby became healthy."* (KDR_04).<br><br>*"Anxiety, fear, worry… we encounter these most often."* (Ka_Ru_Ott_2).<br><br>*"After explanation… slowly they understood… became calmer."* (KDR_03).	role in the success of home-based preterm-infant care and in sustaining continuity of care after discharge.

No	Theme	*Definition Essence*	Domain	Subtheme	Number of References	Example Quotation	Implications for the Research Question
						"Counselling attended by mother and husband." (Nks_Ners_WA_4);<br><br>*"We try to ensure the husband is present for psychological support."* (Nks_Bdn_KIA_3).<br><br>*"Parents don't have the final say… the grandmother intervenes."* (Nks_Ners_WA_5).<br><br>*"We involve community health cadres to keep communication with families."* (Ka_Pkm_8).	
3.	*Health Worker Capacity and Resources*	Individual and institutional capacity of health workers to carry out effective communication, including skills, availability of educational media, time allocation, and supervisory systems that support communication practices.	Meso	a. *Training and Skills*<br>b. *Educational Materials Availability*<br>c. *Workload and Time Constraints*<br>d. *Supervision and Performance Evaluation*<br>e. *SOPs and Protocols*	236 excerpts / 59 participants	*"We provide various trainings… pediatric and maternal nursing…"* (OP_1).<br><br>*"We have a dedicated WhatsApp group… they can ask directly."* (Nks_Bdn_KIA_1);<br><br>"If they need anything, I'm in the next room." (Nks_Ners_WA_8).<br><br>*"More effective through audiovisual."* (Ka_Ru_Ott_2).<br><br>*"We evaluate every activity…"* (Ka_Pkm_4);	Indicates that communication competence and structural support for health workers constitute essential prerequisites for the successful implementation of family-centred communication programmes.

No	Theme	*Definition Essence*	Domain	Subtheme	Number of References	Example Quotation	Implications for the Research Question
						"I often observe how staff communicate." (Ka_Ru_Ott_1).<br><br>*"No standardized SOP… it needs to be developed."* (Nks_SPA_Ott_6).	
4.	*System, Policy, and Access*	Health system, policy, and resource support that facilitates continuous communication across levels of care, including financing mechanisms, inter-agency coordination, referral systems, and geographic accessibility.	Macro	a. *Policy and Regulation*<br>b. *Program Evaluation / Monitoring and Evaluation*<br>c. *Financing and BPJS Administration*<br>d. *Interagency Coordination*<br>e. *Referral System*<br>f. *Resource (Infrastructure, Tools, Supplies)*<br>g. *Geographic and Economic Access*	181 excerpts / 59 participants	*"In health… nothing specific for preterm infants."* (OP_4);<br><br>*"For prematurity specifically… none yet."* (EKS_DKK_5).<br><br>*"Don't design a communication programme without measures of success."* (OP_4).<br><br>*"No mechanism besides the control card…"* (EKS_DKJ_3);<br><br>*"We can't visit all facilities for monitoring."* (EKS_DKJ_5).<br><br>*"With active BPJS, it helps a lot… no need to worry."* (IBP_03).<br><br>*"Without BPJS… referral for preterm infants is very difficult."* (Nks_Bdn_KIA_3).	Indicates that communication effectiveness in preterm-infant care is contingent upon policy support, financing mechanisms, and the availability of resources across multiple levels of the health system.

No	Theme	*Definition Essence*	Domain	Subtheme	Number of References	Example Quotation	Implications for the Research Question
						"We must stay connected… information from the hospital must reach field workers." (OP_3).<br><br>*"We gathered the PKRS team… to enhance communication skills."* (EKS_DKJ_4).<br><br>"Referral from districts uses Sisrute, but there's no referral back." (OP_2).<br><br>*"Sometimes nothing is written in the MCH handbook…"* (Ka_Pkm_8).<br><br>*"Preterm quota is very limited."* (Nks_Bdn_KIA_6).<br><br>*"Many channels… even via Zoom."* (EKS_JKN_1).<br><br>*"No dedicated staff… so it doesn't run."* (EKS_DKJ_4).<br><br>*"The location is far… so no one comes."* (Ka_Ru_WA_3).<br><br>"*In cold areas… afraid the baby will be hypothermic.*" (Nks_Ners_Ott_3).	

No	Theme	*Definition Essence*	Domain	Subtheme	Number of References	Example Quotation	Implications for the Research Question
						"In remote areas, even buying Internet data is difficult." (EKS_DKJ_4).	
