# Supplementary material for: Communication dynamics in the care of preterm infants: a multi-level qualitative study from a high-burden district in Indonesia
Source: Int J Equity Health. 2026 Jun 5;25:193. doi: 10.1186/s12939-026-02914-y (PMC13459254; doi:10.1186/s12939-026-02914-y)
Supplement: Supplementary file 3 [file 12939_2026_2914_MOESM2_ESM.pdf]

Supplementary File 2 – Data Collection Instruments (Indonesian and English Version)

Catatan:
Dokumen ini merupakan versi publik dari panduan wawancara/FGD. Beberapa penyesuaian editorial ringan dilakukan untuk kejelasan dan kesesuaian dengan standar diseminasi akses terbuka. Substansi, struktur, dan tujuan pertanyaan tetap sama dengan versi yang digunakan pada pengambilan data lapangan.
English Version
Note:
This is the public version of the in-depth interview/FGD guide. Minor editorial refinements were made to enhance clarity and alignment with open-access dissemination standards. The substantive content, structure, and intent of the questions remain unchanged from the version used during fieldwork.

Conceptual Orientation of the Data Collection Instruments

The in-depth interview and focus group discussion guides were informed by behavioural and health communication frameworks, including the Integrated Behavioural Model, Health Belief Model, Social Cognitive Theory, Social and Behaviour Change Communication, and the Continuum of Care framework. These frameworks were used as sensitising concepts to inform the focus of inquiry, rather than as prescriptive templates for questioning or analysis.

The guides were intentionally designed to elicit participants' lived experiences and interpretations of communication practices across individual, interpersonal, community, organisational, and system levels. No questions were explicitly framed around theoretical constructs, allowing themes to emerge inductively from the data.

Conceptual and Analytic Mapping of Interview and FGD Domains

Interview / FGD Domain	Focys of inquiry	Level of Analysis	Related Conceptual Orentation
Communication experiences	Information exchange, clarity, timing, and interaction	Individual / Interpersonal	Health communication, SBCC
Barriers to communication	Emotional, cultural, organisational, and systemic constraints	Interpersonal / Organisational	Perceived barriers, system readiness
Information needs	Content, format, and delivery preferences	Individual / Family	Knowledge, perceived benefits
Expectations of ideal communication	Desired practices and support mechanisms	Community / Organisational	Social support, self-efficacy
Policy and regulation	SOPs, referral systems, financing	System	Continuum of care

PANDUAN WAWANCARA MENDALAM (Versi Publik – Bahasa Indonesia)

A. Deskripsi

Panduan wawancara semi-terstruktur ini digunakan untuk wawancara mendalam dengan berbagai kelompok partisipan terkait perawatan bayi prematur. Instrumen ini menggali pengalaman, persepsi, hambatan, serta kebutuhan komunikasi pada lintas level pelayanan. Pendekatan semi-terstruktur memberikan fleksibilitas untuk eksplorasi mendalam sambil menjaga fokus pada tema inti penelitian.

B. Tujuan

1. Menggali pengalaman komunikasi dalam perawatan bayi prematur.
2. Mengidentifikasi hambatan komunikasi yang dialami partisipan.
3. Memahami kebutuhan informasi terkait perawatan bayi prematur di berbagai level sistem kesehatan.

C. Komponen Panduan Wawancara

1. Persiapan
    a. Memastikan alat rekam berfungsi
    b. Menyiapkan tempat yang tenang dan menjaga privasi.
2. Bagian Pembuka
    a. Peneliti memperkenalkan diri dan menjelaskan tujuan wawancara.
    b. Menjelaskan kerahasiaan data dan hak partisipan untuk berhenti kapan saja.
    c. Menjelaskan proses perekaman audio dan dokumentasi.
    d. Menegaskan bahwa tidak ada jawaban benar atau salah.
    e. Meminta izin kontak lanjutan jika diperlukan klarifikasi.

    Contoh pembukaan:

    "Terima kasih telah meluangkan waktu. Wawancara ini bertujuan memahami pengalaman komunikasi dalam perawatan bayi prematur. Seluruh informasi bersifat rahasia dan hanya untuk kepentingan penelitian. Dengan izin Anda, wawancara akan direkam agar data terdokumentasi dengan baik."

D. Pertanyaan Wawancara (General untuk Semua Partisipan)

(Catatan: Hanya gunakan probing bila relevan sesuai dinamika wawancara. Di lapangan probing bersifat opsional dan fleksibel. Pertanyaan yang berkaitan dengan aspek regulasi atau kebijakan tidak diajukan kepada ibu bayi prematur, dan hanya ditujukan bagi informan yang memiliki kewenangan atau pengetahuan terkait.)

1. Pengalaman Komunikasi
    a. Dapatkah Anda menceritakan bagaimana Anda biasanya menyampaikan atau menerima informasi terkait kondisi dan perawatan bayi prematur?

    Probing (opsional):
    - Informasi apa yang dianggap penting?
    - Disampaikan sekaligus atau bertahap?
    - Siapa yang berperan menyampaikan informasi?

- Bagaimana memastikan informasi dipahami?

b. Bagaimana Anda menilai keterampilan komunikasi Anda atau tenaga kesehatan yang terlibat?

   Probing:

   - Faktor yang membuat komunikasi efektif
   - Pengalaman komunikasi yang sulit atau berkesan
   - Hambatan seperti waktu, emosi, atau situasi klinis

c. Saluran komunikasi apa yang biasanya digunakan, dan bagaimana efektivitasnya?

   Probing:

   - Tatap muka, telepon, WhatsApp, media digital
   - Aksesibilitas bagi keluarga
   - Saluran paling efektif dan alasannya

d. Bagaimana ruang diberikan bagi keluarga/partisipan untuk menyampaikan kekhawatiran atau emosi terkait perawatan?

   Probing:

   - Kesempatan bertanya
   - Waktu khusus untuk diskusi
   - Dukungan emosional

2. Hambatan Komunikasi

   Apa hambatan utama yang Anda alami dalam komunikasi terkait kondisi atau tindakan perawatan bayi prematur?

   Probing:

   - Istilah teknis
   - Reaksi emosional keluarga
   - Faktor budaya, lingkungan, atau sistem

3. Kebutuhan Informasi

   Menurut Anda, informasi apa yang paling dibutuhkan dalam perawatan bayi prematur, dan bagaimana sebaiknya disampaikan?

   Probing:

   - Media edukasi: leaflet, gambar, video
   - Protokol atau panduan komunikasi
   - Keseimbangan antara informasi lengkap dan kecemasan keluarga

4. Harapan terhadap Komunikasi

   Menurut Anda, seperti apa komunikasi yang ideal antara tenaga kesehatan, keluarga, dan lintas profesi?

   Probing:

   - Kebutuhan pelatihan komunikasi
   - Pelibatan keluarga dalam keputusan
   - Pengalaman menerima feedback

5. Regulasi dan Kebijakan

Bagaimana pandangan Anda mengenai kebijakan atau standar pelayanan yang mengatur komunikasi dalam perawatan bayi prematur?

Probing:

- Kejelasan SOP komunikasi
- Koordinasi antarprofesi
- Gap antara kebijakan dan praktik
- Rekomendasi perbaikan

E. Bagian Penutup

Contoh penutup:

"Apakah ada hal lain yang ingin Anda tambahkan?"

"Sebelum kita akhiri, apakah ada pernyataan yang ingin diklarifikasi, ditambahkan, atau diperbaiki?"

"Baik, jika tidak ada tambahan, mohon izin data ini akan diproses dalam tahap analisis. Terima kasih atas waktu dan kontribusi Anda."

IN-DEPTH INTERVIEW GUIDE (Public Version – English)

A. Description

This semi-structured interview guide is designed for in-depth interviews with individuals involved in preterm infant care across multiple levels of the health system. It explores experiences, perceptions, barriers, and information needs related to communication. The semi-structured format provides flexibility while maintaining focus on core themes.

While the core domains of inquiry were consistent across participant groups, questions were flexibly adapted in wording and emphasis to reflect participants' roles, responsibilities, and levels of authority. This approach enabled comparative analysis across actors while preserving contextual relevance and depth.

B. Objectives

1. To explore participants' communication experiences in preterm infant care.
2. To identify barriers encountered during communication processes.
3. To understand information needs across care settings.

C. Components of the Interview Guide

1. Preparation
    a. Ensure the recording device functions properly.
    b. Ensure a quiet, private location.
2. Opening

    The interviewer:
    a. Introduces themselves and explains the study purpose.
    b. Explains confidentiality and voluntary participation.
    c. Requests consent for audio-recording.
    d. Emphasises that there are no right or wrong answers.
    e. Requests permission for follow-up clarification if needed.

        Sample introduction:

        "Thank you for taking the time to participate. This interview aims to understand communication experiences in preterm infant care. All information will remain confidential. With your permission, I will record this session to ensure accuracy."

D. Interview Questions (General Across Actor Groups)

(Note: Use probing only when relevant to the flow of the interview. In practice, probes are optional and applied flexibly. Questions related to regulations or policies were not asked of mothers of preterm infants and were directed only to participants with appropriate authority or contextual knowledge.

Probing questions were used to deepen understanding and clarify participants' meanings, supporting inductive exploration rather than steering responses toward predefined theoretical interpretations.)

1. Communication Experiences
    a. Could you describe how you typically communicate or receive information about the care of preterm infants?
       Probing:
       - Key information delivered
       - Delivered at once or gradually
       - Who communicates the information
       - How understanding is ensured
    b. How would you assess the communication skills of yourself or the health providers involved?
       Probing:
       - What makes communication effective
       - Examples of difficult or memorable interactions
       - Emotional or situational barriers
    c. Which communication channels are typically used, and how effective are they?
       Probing:
       - Face-to-face, telephone, WhatsApp, digital media
       - Accessibility for families
       - Most effective channels and reasons
    d. How is space provided for families to express concerns and receive emotional support?
       Probing:
       - Opportunities to ask questions
       - Dedicated time for discussion
       - Approaches to emotional support
2. Communication Barriers
   What challenges do you encounter when explaining clinical conditions or procedures related to preterm care?
   Probing:
   - Medical terminology
   - Emotional reactions
   - Cultural or environmental factors

3. Information Needs
   In your experience, what information is most needed in preterm infant care, and how should it be delivered?
   Probing:
   - Educational tools: leaflets, visuals, videos
   - Use of protocols or communication guidelines

- Balancing completeness and clarity without causing distress

4. Expectations for Communication

   What does ideal communication between health providers, families, and teams look like to you?

   Probing:
   - Need for training or support
   - Family involvement in decision-making
   - Feedback received and responses

5. Regulation and Policy

   How do you view current policies or standards that guide communication in preterm infant care?

   Probing:
   - Clarity of communication SOPs
   - Interprofessional coordination
   - Gaps between policy and practice
   - Recommendations for improvement

E. Closing Section

   Examples:

   “Is there anything else you would like to add?”

   “Before closing, is there anything you would like to clarify or revise?”

   “Thank you for your time. With your permission, this recording will now proceed to the analysis stage.”

PANDUAN FGD (Focus Group Discussion) – Versi Publik (Bahasa Indonesia)

A. Deskripsi

Panduan FGD ini digunakan untuk menggali pandangan kolektif mengenai pengalaman, hambatan, dan kebutuhan komunikasi dalam perawatan bayi prematur dari berbagai kelompok: tenaga kesehatan, manajer layanan, pemegang program, organisasi profesi, dan lembaga pembiayaan. Format semi-terstruktur memastikan diskusi terarah namun tetap fleksibel untuk menghasilkan perspektif lintas-aktor.

B. Tujuan

1. Mengidentifikasi pandangan bersama terkait praktik komunikasi dalam perawatan bayi prematur.
2. Menggali hambatan komunikasi pada level tim, organisasi, dan sistem.
3. Menghimpun rekomendasi dan kebutuhan penguatan komunikasi lintas sektor.

C. Komponen FGD

1. Persiapan
2. Memastikan setup ruang diskusi kondusif (private, suara jelas).
3. Memastikan alat perekam aktif dan posisi duduk mendukung interaksi.
4. Memastikan daftar peserta hadir.

D. Pembukaan

Moderator:

1. Memperkenalkan diri dan tim.
2. Menjelaskan tujuan FGD.
3. Menyampaikan aturan dasar: saling menghargai, tidak memotong, tidak ada jawaban benar/salah, kerahasiaan terjamin.
4. Meminta izin untuk merekam audio.
5. Menyampaikan harapan kontribusi setara dari seluruh anggota.

   Contoh pembukaan:

   “Terima kasih telah hadir. Diskusi ini bertujuan memahami pengalaman dan tantangan komunikasi dalam perawatan bayi prematur. Tidak ada jawaban benar atau salah. Semua pendapat penting. Dengan izin Anda, FGD akan direkam agar datanya akurat.”

E. Pertanyaan FGD (General untuk Semua Kelompok Aktor)

(Catatan: Hanya gunakan probing bila relevan sesuai dinamika wawancara. Di lapangan probing bersifat opsional dan fleksibel. Pertanyaan yang berkaitan dengan aspek regulasi atau kebijakan tidak diajukan kepada ibu bayi prematur, dan hanya ditujukan bagi informan yang memiliki kewenangan atau pengetahuan terkait.)

1. Pengalaman Komunikasi Lintas Tim / Lintas Level
   a. Bagaimana pengalaman Anda sebagai bagian dari tim / fasilitas / lembaga dalam berkomunikasi dengan keluarga bayi prematur serta antar-profesi?
      Probing:

- Informasi apa yang wajib disampaikan dan oleh siapa?
- Apakah penyampaian dilakukan berjenjang?
- Bagaimana memastikan pesan konsisten antar-profesi?
- Contoh praktik komunikasi yang berjalan baik / kurang optimal.

2. Mekanisme dan Saluran Komunikasi
   Saluran komunikasi apa yang paling sering digunakan dalam interaksi dengan keluarga dan antar-fasilitas? Bagaimana efektivitasnya?
   Probing:
   - Tatap muka, WhatsApp, telepon, rekam medis, rujukan digital / SISRUTE
   - Apakah semua saluran mudah diakses semua pihak?
   - Apakah ada standar komunikasi antarprofesi?
   - Konsistensi pesan antara tenaga kesehatan primer–rujukan–fasilitas lanjutan.
3. Hambatan Komunikasi (Tim, Organisasi, Sistem)
   Apa hambatan utama yang Anda hadapi dalam komunikasi terkait perawatan bayi prematur?
   Probing:
   - Waktu terbatas, beban kerja
   - Penggunaan istilah teknis
   - Ketidaksinkronan informasi antarprofesi
   - Perbedaan persepsi dalam tim
   - Keterbatasan media edukasi
   - Tantangan budaya / sosial keluarga
   - Hambatan sistem (SOP, rujukan, kebijakan, pembiayaan)

4. Kebutuhan Informasi dan Media
   Menurut Anda, informasi apa yang paling dibutuhkan keluarga dan tenaga kesehatan untuk memastikan kelanjutan perawatan bayi prematur?
   Probing:
   - Konten inti yang wajib disampaikan
   - Media edukasi (leaflet, buku, poster, video, aplikasi)
   - Ketersediaan dan kesesuaian SOP komunikasi
   - Integrasi edukasi antara fasilitas primer–rujukan–komunitas
5. Harapan terhadap Komunikasi Ideal
   Menurut Anda, seperti apa komunikasi ideal dalam perawatan bayi prematur dari sisi tim, fasilitas, dan sistem?
   Probing:
   - Kebutuhan pelatihan komunikasi
   - Mekanisme koordinasi efektif antarprofesi
   - Peran keluarga dalam pengambilan keputusan

- Sistem yang mendukung komunikasi (SOP, rujukan, BPJS, pemantauan pascapulangan)

6. Kebijakan dan Regulasi

   Bagaimana pandangan Anda mengenai kebijakan dan standar komunikasi dalam perawatan bayi prematur? Apa yang masih perlu diperbaiki?

   Probing:

   - SOP komunikasi internal
   - Informasi rujukan
   - Prosedur discharge planning
   - Standar BPJS terkait administrasi dan klaim
   - Kesenjangan antara kebijakan tertulis dan praktik lapangan

F. Penutup

a. Moderator merangkum poin utama diskusi.
b. Mengundang peserta menambahkan hal yang belum dibahas.
c. Memberikan kesempatan klarifikasi.

   Contoh penutup:

   "Sebelum kita akhiri, apakah ada yang ingin ditambahkan, diklarifikasi, atau diperbaiki? Jika tidak ada, terima kasih atas kontribusinya. Semua masukan sangat berharga untuk analisis penelitian dan pengembangan model komunikasi."

FGD GUIDE (Public Version – English)

A. Description

This semi-structured FGD guide explores collective experiences, communication practices, barriers, and expectations across professional groups involved in preterm infant care (health workers, facility managers, health offices, professional bodies, and insurers). The guide maintains consistency while allowing flexible group dynamics.

While the core domains of inquiry were consistent across participant groups, questions were flexibly adapted in wording and emphasis to reflect participants' roles, responsibilities, and levels of authority. This approach enabled comparative analysis across actors while preserving contextual relevance and depth.

B. Objectives
   1. To explore shared communication experiences in preterm care.
   2. To identify communication barriers across teams and system levels.
   3. To gather cross-sector recommendations for strengthening communication.

C. FGD Components
   1. Preparation
      a. Ensure recording devices work.
      b. Arrange seating to promote interaction.
      c. Confirm participant attendance.
   2. Opening
      Moderator:
      a. Introduces self and research purpose.
      b. Explains confidentiality and voluntary participation.
      c. Sets ground rules (respect, no interruptions, no right/wrong answers).
      d. Requests consent for audio-recording.
         Opening statement:
         "Thank you for joining this discussion. We aim to understand communication challenges and strengths in preterm infant care. Your contributions are confidential and equally important. With your permission, we will record this discussion to ensure accuracy."

D. FGD Questions (General Across Actor Groups)

(Note: Use probing only when relevant to the flow of the interview. In practice, probes are optional and applied flexibly. Questions related to regulations or policies were not asked of mothers of preterm infants and were directed only to participants with appropriate authority or contextual knowledge.

Probing questions were used to deepen understanding and clarify participants' meanings, supporting inductive exploration rather than steering responses toward predefined theoretical interpretations.)

1. Communication Experiences
   Could you describe how your team or institution typically communicates with families and other facilities regarding preterm infant care?
   Probing:
   - Key messages and responsible personnel
   - Gradual vs. one-time information delivery
   - Ensuring consistency of messages across teams
   - Examples of effective or challenging communication
2. Communication Channels
   What communication channels are commonly used, and how effective are they?
   Probing:
   - Face-to-face, calls, WhatsApp, digital systems
   - Accessibility for families
   - Standardised communication pathways
   - Referral communication between PHC–hospitals–community
3. Barriers
   What are the main communication barriers encountered at clinical, organisational, or system levels?
   Probing:
   - Time pressure
   - Medical terminology
   - Emotional reactions of families
   - Interprofessional misalignment
   - Limited educational materials
   - Systemic issues (SOPs, referral pathways, financing)
4. Information Needs
   What information do families and health workers most need, and how should it be delivered?
   Probing:
   - Educational tools
   - Protocols or communication standards
   - Consistency across the continuum of care
5. Expectations
   What does ideal communication in preterm infant care look like at team and system levels?
   Probing:
   - Communication skills training
   - Coordination mechanisms
   - Family-centred decision-making
   - Use of structured tools or SOPs
6. Policy and Regulation

How do current policies support or hinder communication in preterm infant care?
Probing:

- Written standards
- Referral mechanisms
- Differences between policy and practice
- Recommendations for improvement

E. Closing
Examples:
"Is there anything else you would like to add?"
"Would you like to clarify or revise any earlier statements?"
"Thank you for your contributions. Your insights will meaningfully support our analysis and model development."

LEMBAR OBSERVASI PARTISIPATIF (INONESIAN VERSION)

A. DESKRIPSI

Lembar observasi partisipatif ini dirancang untuk mencatat pola komunikasi verbal dan non-verbal antara tenaga kesehatan dan keluarga bayi prematur di NICU atau saat discharge planning. Observasi ini bertujuan untuk memahami konteks sosial-budaya, pola interaksi, serta dinamika komunikasi yang terjadi selama proses asuhan bayi prematur.

B. **INFORMASI UMUM**

1. Tanggal Observasi : (…………isi tanggal pelaksanaan observasi……...)
2. Waktu Observasi : (…………isi waktu pelaksanaan observasi……….)
3. Lokasi : (..isi lokasi pelaksanaan observasi, misal NICU dll.)
4. Nama Pengamat : (...isi nama pengamat yang melakukan observasi…)
5. Nama Partisipan : (..nama atau kode anonim partisipan yang diamati..)

C. ASPEK YANG DIAMATI

1. Pola Interaksi Verbal:
   – Apakah tenaga kesehatan menggunakan bahasa yang sederhana atau teknis?
   – Bagaimana cara tenaga kesehatan menjelaskan informasi kepada keluarga?
   – Bagaimana respons keluarga terhadap informasi yang disampaikan?
   – Apakah mereka bertanya atau hanya mendengarkan?
2. Pola Interaksi Non-Verbal:
   – Apa ekspresi wajah tenaga kesehatan saat menyampaikan informasi (ramah, serius, atau netral)?
   – Bagaimana bahasa tubuh keluarga (misalnya, menunjukkan tanda-tanda ketidakpahaman, kebingungan, atau penerimaan)?
   – Apakah ada kontak mata antara tenaga kesehatan dan keluarga?
3. Konteks Sosial-Budaya:
   – Apakah ada faktor budaya atau kebiasaan lokal yang memengaruhi komunikasi?
   – Apakah konteks sosial (misalnya, jumlah orang dalam ruangan atau situasi emosional) memengaruhi interaksi antara tenaga kesehatan dan keluarga?
4. Pesan dan Media Komunikasi yang Digunakan
   – Apakah pesan yang disampaikan relevan dengan kebutuhan.
   – Media komunikasi apa saja yang digunakan untuk menyampaikan informasi (misalnya, brosur, gambar, video, atau presentasi digital)?
   – Apakah media yang digunakan mudah dipahami dan sesuai dengan kebutuhan keluarga?

- Bagaimana kualitas visual dan keterbacaan materi yang disajikan?
- Apakah tenaga kesehatan memberikan penjelasan tambahan mengenai media yang digunakan?

5. Respons Keluarga terhadap Informasi yang Disampaikan oleh Tenaga Kesehatan
   - Bagaimana reaksi verbal keluarga saat menerima informasi? Apakah mereka mengajukan pertanyaan atau memberikan tanggapan?
   - Apakah keluarga menunjukkan tanda-tanda pemahaman atau kebingungan terhadap informasi yang diberikan?
   - Sejauh mana keluarga tampak proaktif dalam meminta klarifikasi atau informasi tambahan?
   - Bagaimana respons emosional keluarga, seperti antusiasme, kekhawatiran, atau keraguan terhadap informasi tersebut?

D. CATATAN OBSERVASI

1. Deskripsi Interaksi:

(Tuliskan deskripsi rinci tentang interaksi yang diamati, termasuk dialog, reaksi, dan konteks situasional):<br>**Catatan :**

2. Refleksi Pengamat:

(Tuliskan refleksi pribadi tentang apa yang diamati, termasuk kesan dan interpretasi terhadap pola komunikasi) :<br>**Catatan :**

3. Tindak Lanjut (Apakah ada rekomendasi untuk perbaikan komunikasi berdasarkan observasi ini?) :

(Tuliskan saran atau rekomendasi untuk meningkatkan efektivitas komunikasi antara tenaga kesehatan dan keluarga)<br>**Catatan :**

LEMBAR OBSERVASI PARTISIPATIF (INONESIAN VERSION)

A. DESKRIPSI

This participatory observation form is designed to document verbal and non-verbal communication patterns between health workers and families of preterm infants in the NICU or during discharge planning. The aim is to capture the socio-cultural context, interaction patterns, and communication dynamics that occur throughout the care process of preterm infants.

B. GENERAL INFORMATION

Observation Date	: (…insert date of observation…)
Observation Time	: (…insert time of observation…)
Location	: (…insert observation site, e.g., NICU…)
Observer Name	: (…insert name of the observer…)
Participant Name/Code	: (…insert participant name or anonymised code…)

C. ASPECTS OBSERVED

1. Verbal Interaction Patterns
   a. Did the health worker use simple language or technical terminology?
   b. How did the health worker deliver information to the family?
   c. How did the family respond to the information provided?
   d. Did they ask questions or mostly listen?
2. Non-Verbal Interaction Patterns
   a. What facial expressions did the health worker show while communicating (friendly, serious, neutral)?
   b. What family body language was observed (signs of confusion, uncertainty, acceptance)?
   c. Was there eye contact between the health worker and the family?
3. Socio-Cultural Context
   a. Were there cultural or local practices influencing the communication process?
   b. Did the social environment (e.g., number of people in the room, emotional situation) affect interactions between health workers and families?
4. Messages and Communication Media Used
   a. Were the messages delivered relevant to the family’s needs?
   b. What communication media were used (e.g., brochures, pictures, videos, digital presentations)?
   c. Were the materials understandable and appropriate for the family?
   d. How was the visual quality and readability of the materials?

e. Did the health worker provide additional explanation regarding the media used?

5. Family Responses to Information Provided by Health Workers
    a. How did the family verbally react to the information (questions, comments)?
    b. Did they show signs of understanding or confusion?
    c. To what extent did they appear proactive in seeking clarification or additional information?
    d. What emotional responses were observed (enthusiasm, worry, uncertainty)?

D. Observation Notes

1. Interaction Description:

(Write a detailed description of the interaction observed, including dialogue snippets, reactions, and contextual factors.)<br>***Notes:***

2. Observer Reflection:

(Write your personal reflection on the observed interaction, including interpretations and impressions of communication patterns.)<br>Notes:

KERANGKA ANALISIS DOKUMEN (VERSI INDONESIA)

A. DESKRIPSI

Kerangka analisis dokumen dirancang untuk mengevaluasi materi tertulis yang relevan dengan komunikasi dalam asuhan bayi prematur. Dokumen yang dianalisis dapat mencakup panduan discharge planning, catatan medis, materi edukasi, dan dokumen kebijakan terkait perawatan bayi prematur. Analisis ini bertujuan untuk memahami bagaimana informasi disampaikan kepada keluarga bayi prematur dan sejauh mana materi tersebut memenuhi kebutuhan mereka.

B. TUJUAN

1. Menilai kelengkapan informasi yang disampaikan kepada keluarga bayi prematur.
2. Mengidentifikasi bahasa yang digunakan dalam dokumen (apakah sederhana atau teknis).
3. Menilai relevansi materi terhadap kebutuhan informasi keluarga.

C. KOMPONEN KERANGKA ANALISIS DOKUMEN

1. Kelengkapan Informasi
   a. Apakah semua aspek penting dari perawatan bayi prematur tercakup dalam dokumen?
   b. Contoh: stabilisasi suhu, nutrisi, tanda bahaya, imunisasi, dan stimulasi perkembangan.
   c. Apakah ada informasi yang kurang atau tidak jelas?
2. Bahasa yang Digunakan
   a. Apakah bahasa yang digunakan mudah dipahami oleh keluarga?
   b. Contoh: penggunaan istilah medis yang mungkin sulit dipahami oleh orang awam.
   c. Apakah ada istilah teknis yang perlu dijelaskan lebih lanjut?
3. Relevansi Materi
   a. Apakah materi yang disampaikan sesuai dengan kebutuhan informasi keluarga?
   b. Contoh: apakah materi tersebut membantu keluarga memahami perawatan bayi prematur di rumah?
   c. Apakah ada umpan balik dari keluarga terkait materi yang diberikan?
4. (Khusus dokumen kebijakan / regulasi).
   Bagaimana Dukungan dan hambatan kebijakan / regulasi asuhan bayi prematur dalam konteks komunikasi.

D. METODE PENGUMPULAN DATA

Identifikasi Dokumen: Mengumpulkan dokumen yang relevan dari rumah sakit (panduan *discharge planning*, catatan medis), puskesmas (materi edukasi), dan organisasi profesi (pedoman kebijakan).

No	Dokumen	Kelengkapan Informasi	Bahasa yang Digunakan	Relevansi Materi	Komentar
contoh	Panduan Discharge Planning	Lengkap / Tidak lengkap	Sederhana / Teknis	Relevan / Tidak Relevan	Informasi tentang tanda bahaya belum dijelaskan secara rinci
1.					
2.					
3.					
Dst.					

DOCUMENT ANALYSIS FRAMEWORK (English Version)

A. DESCRIPTION

This document analysis framework is designed to evaluate written materials relevant to communication within preterm infant care. Documents may include discharge planning guidelines, medical records, educational materials, and policy documents related to preterm infant care. The purpose of the analysis is to examine how information is communicated to families and to assess the extent to which these materials meet their needs.

B. OBJECTIVES

1. To assess the completeness of information provided to families of preterm infants.
2. To identify the level of language used in the documents (simple vs. technical).
3. To evaluate the relevance of the materials to the information needs of families.

C. COMPONENTS OF THE DOCUMENT ANALYSIS FRAMEWORK

1. Completeness of Information
   a. Do the documents cover all essential aspects of preterm infant care?
   b. Examples: temperature stabilisation, nutrition, danger signs, immunisation, developmental stimulation.
   c. Are there any missing, unclear, or insufficiently detailed sections?
2. Language Used
   a. Is the language understandable for families?
   b. Example: the use of medical terms that may be unfamiliar to non-medical audiences.
   c. Are there technical terms requiring additional explanation?
3. Relevance of the Materials
   a. Does the content meet the information needs of families?
   b. Example: whether the materials help families understand how to care for a preterm infant at home.
   c. Is there any feedback from families regarding the usefulness or clarity of the materials?
4. (For policy or regulatory documents only)
   Assessment of how policies and regulations support or hinder communication within preterm infant care.

D. DATA COLLECTION METHOD

Collect relevant documents from:

1. Hospitals (e.g., discharge planning guidelines, medical records)
2. Primary health centres (educational materials)
3. Professional bodies (policy and clinical guidelines)

No	Dokumen	Kelengkapan Informasi	Bahasa yang Digunakan	Relevansi Materi	Komentar
example	Discharge Planning guideline	Complete/ incomplete	Simple/technical	Relevant / not Relevant	Danger sign not explained in sufficient detail
1.					
2.					
3.					
4. Dst.					
